# Supplementary material for: Post-Vaccination Streptococcus pneumoniae Carriage and Virulence Gene Distribution among Children Less Than Five Years of Age, Cape Coast, Ghana
Source: Microorganisms. 2020 Dec 13;8(12):1987. doi: 10.3390/microorganisms8121987 (PMC7764876; doi:10.3390/microorganisms8121987)
Supplement: Supplementary file 1 [file microorganisms-08-01987-s001.pdf]

Supplementary table

Table S1. Oligonucleotides used for molecular genotyping of pneumococcal strains.

Primer No.	Primer name	Primer sequence (5′ to 3′)
	RD10-*psrP* (positive)	
722	RD10-*psrP*-F	5′- CAACTGCTAAGAAGGTCGAAG-3′
723	RD10-*psrP*-R	5′- CAAGTATACCCACTACCCTGC-3′
	RD10-*psrP* (negative)	
724	RD10-Neg-F	5′- CACCCTTCTTCAACTCCTACG-3′
725	RD10-Neg-R	5′- CTTCCAAGCGAGCCACCATCC-3′
	pavB	
161	Fnbmut3-F	5′- GCTACCAAATACTGGTGAGACTC-3′
254	TCS08mut6-R	5′- CTCCTATTTGAACTTCATGACTAC-3′
	Pilus islet-1(positive)	
279	*rrgB*mut1-F	5′- GTGGTATCGGAATGTTGCC-3′
280	*rrgB*mut2-R	5′- GATCAATATTCACTCCTAGAG-3′
	Pilus islet-1 (negative)	
287	PI-1-F	5′- CGCCTTGGATGCATTGAGC-3′
288	PI-1-R	5′- GTATTACAAGATATTATTTCACC-3′
	Pilus islet-2 (positive)	
301	*pitB*mut1-F	5′- GAGTGTCTGGGGAGAATTCCTTTAC-3′
302	*pitB*mut1-R	5′- GGTTATTGCTGAATTAGGATCCGC-3′
	Pilus islet-2 (negative)	
305	PI-2-F	5′- AAAATCAGCAGTATCCATGTG-3′
306	PI-2-R	5′- GGGACTGGAAAACTTATTAGC-3′
857	*lytA*-F	5′- GAAATTAATGTGAGTAAATTAAGAACAG-3′
859	*lytA*-R	5′- TTTTACTGTAATCAAGCCATCTGGC-3′
1686	*pcpA*_F	5′- CTCCTAGTTCGGAAGTAATCA-3′
1687	*pcpA*_R	5′- CTTGACCAGCTTCAAACTCTA-3′
